# Perspectives on Alcohol Taxation

**Published:** 1996

**Authors:** Donald Kenkel, Willard Manning

**Affiliations:** Donald Kenkel, Ph.D., is an associate professor in the Department of Consumer Economics and Housing, Cornell University, Ithaca, New York. Willard Manning, Ph.D., is a professor in the Institute for Health Services Research, School of Public Health, University of Minnesota, Minneapolis, Minnesota

**Keywords:** sales and excise tax, public policy on AOD, AOD consumption, AOD price, public health, economic cost of AODU (alcohol and other drug use), social cost of AODU, socioeconomic status, employment

## Abstract

The issue of alcohol taxation can be viewed from several angles: public health, revenue generation, economic efficiency, fairness, and effects on employment. Conclusions about when an alcohol tax increase is appropriate or effective—or by how much a tax should be increased—differ widely, however, depending on which of these perspectives is taken. Policymakers trying to find a balance among the different perspectives must weigh the multiple trade-offs involved when a tax increase is proposed. Considerations include how different drinking populations respond to tax-induced higher alcohol prices, the equity of a tax for all members of society, and the effects of displacement for workers in alcohol-related industries.

Except during Prohibition, the taxation of alcohol has been an enduring part of U.S. fiscal policy. Early in the Nation’s history, Alexander Hamilton, the first Secretary of the Treasury, proposed a whiskey tax to help pay off debts from the Revolutionary War. In support of Hamilton’s proposal, the Philadelphia College of Physicians proffered a combined professional opinion that “a great proportion of the most obstinate, painful, and mortal disorders which affect the human body are produced by distilled spirits” (Slaughter 1986). Congress was persuaded to enact a Federal alcohol tax in 1791, but enforcement met with some resistance. In what became known as the Whiskey Rebellion of 1794, about 500 armed farmers in the back country of western Pennsylvania attacked Federal tax collectors in opposition to the tax on the plentiful amounts of whiskey they distilled from their corn crops. The farmers’ rebellion against the tax fizzled when President George Washington ordered troops into the area, however, and the tax stood.

Alcohol taxation remains controversial today, although not to the extent of armed rebellion. Many people arrive at different conclusions about whether and to what extent alcohol taxation is appropriate, depending on which of the following perspectives they take on the issue:

- *Public health*. Contemporary medical science confirms that alcohol abuse^1^ is a significant public health problem, even if current warnings are not quite as dire as that given by the Philadelphia College of Physicians.
- *Revenue generation*. Hamilton’s argument that taxes on alcoholic beverages are an attractive source of revenue continues to echo in many State legislatures and congressional hearings.
- *Economic efficiency*. Because problem drinkers^2^ impose costs on others, alcohol taxation may be required to achieve an efficient allocation of resources in society.
- *Equity*. Many people question the fairness of alcohol taxation that falls more heavily on one population group (e.g., the poor) than on another.
- *Employment*. The desirability of alcohol taxation also comes into question because it imposes costs on the workers who produce, distribute, and sell alcoholic beverages.

Recent research by economists interested in alcohol policy can inform each of these diverse perspectives on alcohol taxation. A fundamental question economists examine is how patterns of alcohol consumption and alcohol problems respond to changes in beverage prices and tax rates. The answer to this question clarifies the potential of alcohol taxation as a measure to improve public health and has important implications for tax-revenue generation as well. A smaller, but growing, body of research uses economic theory and available empirical estimates to analyze alcohol taxes from the efficiency and equity perspectives. Other economics research sheds light on the impact taxation has on employment both in alcohol-related industries and on a national level.

After discussing how alcohol consumption responds to taxation and price changes, this article reviews research related to each perspective on alcohol taxes. Although economists do not claim to be able to calculate the “right” tax rate scientifically, the findings and analysis reviewed in this article furnish useful information as society grapples with the trade-offs involved in choosing appropriate alcohol taxes. Cook (1991) and Cook and Moore (1993*b*) provide further details on the economic arguments underlying alcohol taxation for readers interested in more information.

## Effects of Price Changes on Alcohol Consumption

Differences in alcoholic beverage prices and taxes over time and across States have supplied, in effect, a series of “natural experiments” on the price-consumption relationship. Ideally, researchers could compare alcohol consumption among people encountering different alcohol prices but who are otherwise identical. The data provided by natural policy experiments fall short of this ideal, however, because other differences across States, such as average income and education, may be correlated with alcohol price. To overcome the limitations of natural policy experiments, economists have used econometric models (i.e., multivariate statistical analyses guided by economic theory). To the extent that econometric models control statistically for other factors that influence drinking, the remaining differences in drinking can be attributed to variations in alcohol prices. Econometric studies find that alcohol consumption is lower when prices are higher, confirming that the economic “law of demand”—which states that as the price of a good or service rises, consumption of that good or service falls—also applies to alcoholic beverage consumption (for reviews, see Leung and Phelps 1993 and Chaloupka 1993).

The degree to which alcohol consumption is affected by price (i.e., its *price responsiveness*) can be summarized by the price elasticity of demand, which is defined as the percent change in the quantity demanded that results from a 1-percent change in price. The price elasticity of demand for a good partly depends on consumers’ willingness to increase their purchases of substitute goods and services when the price rises. Price elasticities are near zero for goods with few close substitutes (e.g., the price elasticity of medical care is believed to be around −0.2 and that of cigarettes is between −0.4 and −0.6), whereas goods with price elasticities further away from zero indicate that many consumers find acceptable substitutes.

Estimates of the degree of price responsiveness of alcohol consumption vary from study to study. For example, in 15 studies that used data on consumption aggregated at the State or national level, estimated price elasticities for beer ranged from −0.12 to −1.07 (Leung and Phelps 1993). In terms of policy implications, this range of estimates means that a tax increase that raises alcohol prices by 1 percent might reduce beer consumption by as little as 0.12 percent or as much as 1.07 percent. A price elasticity of −1.07 for beer suggests that a substantial portion of consumers consider other substitutes (e.g., nonalcoholic beverages) acceptable.

Although economists agree that alcohol consumption is price responsive, they do not concur about the precise degree of responsiveness. Narrowing the range of plausible price-responsiveness estimates will require improvements in data quality and careful econometric analysis. An important advance over the past 10 to 15 years has been the use of alcohol consumption data gathered from surveys of individuals (i.e., microdata), rather than consumption data aggregated on a State or national level. The price-elasticity estimates from several studies which used micro-data suggest that alcohol consumption is more price responsive than often indicated in analyses of aggregate data (Leung and Phelps 1993).

Economists also have addressed the issue of whether responsiveness to price varies by consumption level. Typically, econometric studies yield an estimate of the average price responsiveness across consumption levels, which might not reliably demonstrate how different subgroups (i.e., light, moderate, and heavy drinkers^3^) respond. For example, persuasive evidence exists that heavy drinkers are price responsive, contrary to what might be expected. Cook and Tauchen (1982) found a significant relationship between liquor taxes and the mortality rate from liver cirrhosis, a reliable proxy for chronic heavy drinking, because death rate from the disease is related to large intakes of alcohol. This finding is supported by evidence (reviewed in the next section) that higher alcohol prices or taxes also reduce other adverse health and safety consequences, such as traffic fatalities.

**Figure 1:**
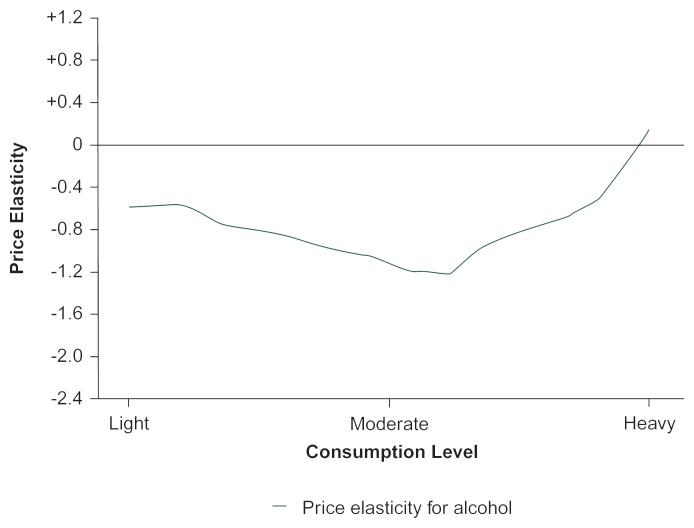
Responsiveness to alcohol price (summarized by price elasticity) varies according to consumption level. Moderate drinkers have an estimated price elasticity of −1.19. Both lighter and heavier drinkers have price elasticities that are closer to zero, meaning that drinkers in these categories are less responsive to price. At the extreme, very heavy drinkers actually appear to increase their consumption as alcohol price increases (i.e., the price elasticity becomes positive), but this estimate is not significantly different from no response to price. NOTE: Drinking levels were defined based on the daily average alcohol consumption for the sample. Moderate drinkers were defined as those who drank at a level approximating the daily average; heavy drinkers consumed alcohol in a range above the average, and light drinkers drank in a range below the average. SOURCE: Manning et al. 1995.

Manning and colleagues (1995) developed an econometric approach to provide a more complete picture of the degree of price responsiveness for subgroups of drinkers. Based on data from the 1983 National Health Interview Survey, respondents were defined as light, moderate, or heavy drinkers compared with the average daily consumption in the sample.^4^ As a result of their analysis, the researchers estimated that moderate drinkers are most responsive to price (see figure 1). Furthermore, heavy drinkers at the extreme end of the scale may be the least responsive to price, although the hypothesis that their demand is altogether unresponsive cannot be rejected.

Kenkel (1996) took a different approach to developing estimates of price elasticity that distinguished between heavy and moderate drinking (defined for the purposes of this study as drinking five or more drinks per occasion or less than five drinks per occasion, respectively). This study focused on estimating the relationship between alcohol prices and various measures of consumption. (For information on the research methodology used, see Kenkel 1996.)

Drawing on data from the 1985 Health Interview Survey, Kenkel (1996) also tested whether people who are better informed about the health consequences of heavy drinking consume less alcohol and respond differently to alcohol prices than those who are less informed. The analysis resulted in an estimate of −0.78 as the average price elasticity of moderate drinking. Heavy drinking also was estimated to be fairly price responsive on average, with an estimated price elasticity of −0.52 for males and −1.29 for females. Evidence emerged of an interaction effect, whereby better informed consumers showed greater price responsiveness. At one extreme, with an estimated price elasticity of −1.65, heavy drinking by the most informed consumers was much more price responsive than was moderate drinking. At the other extreme, the estimated price elasticity of heavy drinking by the least informed consumers was not significantly different from zero.

The price-information interaction effect estimated by Kenkel may reflect the same phenomenon found by Manning and colleagues (1995), because the least informed consumers in Kenkel’s study were, on average, also very heavy drinkers (i.e., females reporting more than 46 days in the past year on which they consumed five or more drinks and males reporting more than 68 days). Both studies suggest that a subset of these drinkers do not respond to price, although this phenomenon may be attributed to an effect of consumer information, not the level of consumption per se. The price-information interaction is consistent with the argument that health education programs will have synergistic effects with other policies to regulate alcohol consumption (Hochheimer 1981).

### Responsiveness of Alcohol Consumption to Taxation

Although the studies cited thus far are limited to price responsiveness, other studies (Cook and Tauchen 1982; Mullahy and Sindelar 1994) provide direct evidence that problem drinking is tax responsive as well. In general, however, the link between alcohol taxes and alcohol prices requires further study. Several issues complicate the tax-price interaction. For one, Federal alcohol taxes in the United States are excise taxes, which means that for each sale of a given quantity of alcoholic beverage, a flat tax amount is due. (Thus, an excise tax is based on the quantity purchased, whereas a sales tax, in contrast, is based on the price of the purchased good.) In what economists term “perfectly competitive” industries, excise taxes are expected to be passed in full to consumers (i.e., a 1-percent excise tax would result in a 1-percent addition to the price that consumers pay). Instead of being perfectly competitive, however, the alcoholic beverage industry is better described as oligopolistic (i.e., a few large firms account for a substantial share of industry sales).

Various analyses suggest that a 1-percent tax increase in oligopolistic industries may increase prices by less than or more than 1 percent (Cook and Moore 1993*b*): Consumers may see prices rise by less than the tax increase if an oligopolistic firm is reluctant to pass on the tax increase because it fears losing market share to competitors who do not likewise increase their prices. Alternatively, if each firm expects its competitors to raise prices after a tax increase, the tax will be passed to consumers. Cook and Moore (1993*b*) suggest the possibility, however, that consumers may even see prices rise in excess of the tax increase if oligopolistic firms use a tax increase to help them coordinate an industrywide price increase. Predicting the likely outcome among these possible scenarios depends on which description of oligopolistic behavior is correct, and this question has not been settled.

Another issue related to excise taxes is that the price of the same alcoholic beverage can differ widely within a geographic area, whereas the tax amount stays the same (Treno et al. 1993). Such price variation is consistent with standard economic price theory, because a can of beer sold at a grocery store is not really the same product as the same beer served in a restaurant or bar. However, variation in the price of alcoholic beverages raises the possibility that the same excise tax is passed to consumers at different rates, depending on where they purchase the beverage.

## Alcohol Taxation From a Public Health Perspective

In light of its goal of reducing harm (i.e., morbidity and mortality) among the population, the public health community finds taxes on alcohol an appealing idea. To the extent that changes in taxation can alter patterns of alcohol consumption that cause or increase morbidity and mortality, higher taxes can serve as a strategy to reduce alcohol-influenced health problems among the public. This strategy involves a subtle but important shift in purposes: The task is not to influence alcohol consumption as a goal in itself, but to reduce certain types of alcohol-related problem behaviors and their consequences. Thus, from the public health perspective, the relevant variable is not the price elasticity of total alcohol consumption or the quantity or frequency of drinking; instead, the key for public health is the price elasticities of drinking and driving, violence, and other alcohol-related problems (i.e., the percent change in occurrence of these problems that results from a 1-percent change in price).

Chaloupka (1993) reviews studies of the relationship between alcohol taxes and prices and various measures of alcohol abuse, including motor vehicle fatality rates, liver cirrhosis death rates, and workplace accidents. These studies consistently indicate that high prices and taxes are associated with fewer alcohol-related problems. These findings do not necessarily contradict the evidence that very heavy drinkers are not responsive to price, because light and moderate drinkers account for nearly one-half of all alcohol-related accidents (Manning et al. 1995). Although moderate drinkers may consume a moderate *average weekly* amount of alcohol, many who experience an accident are bingeing (i.e., compressing their weekly consumption into a small interval of time).

Using various data sets and measures of alcohol-related problems, recent research generally supports earlier findings that alcohol-related problems are price responsive. This research includes several studies of drunk driving that examined microdata on self-reported drunk-driving incidents instead of State-level aggregate data on traffic fatalities.^5^ Analyzing micro-data from the 1985 Health Interview Survey, Kenkel (1993) estimated that the elasticity of drunk driving with respect to the price of alcohol was −0.74 for males and −0.81 for females. In another study, Mullahy and Sindelar (1994) used microdata from the 1983 Health Interview Survey to estimate that the elasticity of drunk driving with respect to the beer tax was −1.9 for males and −1.4 for females. Both studies also provided evidence that drunk driving can be reduced by stricter deterrence policies that increase the certainty and severity of punishment for drunk driving.

Sloan and colleagues (1994*b*) reevaluated the relative effects of alcohol prices and other policies to reduce drunk driving. Using aggregate State-level data from 1982 to 1990, the researchers estimated that alcohol price had a statistically significant effect on motor vehicle fatalities for youth ages 18 to 20, but not for older groups. Interestingly, the effect of price on fatalities was attenuated where the minimum legal drinking age was 21, compared with its stronger effect in States where the drinking age remained at 18 until the mid-1980’s.

In another study, Sloan and colleagues (1994*a*) estimated the relationship between alcohol prices and alcohol-related mortality, including homicides, suicides, accidents, and deaths from alimentary-tract cancers as well as motor vehicle crashes. Statistically significant estimates suggest that higher alcohol prices reduce suicide and cancer death rates. Although some of the researchers’ estimated econometric models also implied that higher alcohol prices reduce traffic deaths, the researchers did not consistently find statistically significant effects with this variable. In several cases, the researchers’ results were less supportive of strong price effects when their econometric models controlled for States’ use of other alcohol policies. These results raise some concern about previous studies that used State-level data but were unable to control for a wide range of State policies.

An intriguing line of research investigates the link between alcohol taxation and another alcohol-related problem: violent crime. Cook and Moore (1993*a*) analyzed State-level data on annual rates of homicide, rape, robbery, and assault for the period 1979 to 1988. In several cases, the study found small but statistically significant effects of higher taxes. The results imply that a 1-percent increase in alcohol taxes reduces rape and robbery by about 0.1 percent. Other results of this study show a strong positive relationship between alcohol consumption and the rates of rape, assault, and robbery. Because controlling for differences in other crime determinants is difficult, however, these estimated relationships do not necessarily indicate that heavy alcohol consumption *causes* high crime rates. Still, the link between alcohol consumption and violent crime, coupled with the link between prices and alcohol consumption, suggests that this line of research holds promise.

## Alcohol Taxation From a Revenue Generation Perspective

As Hamilton foresaw in the 18th century, taxes on alcohol are an enticing revenue generator. Current Federal excise tax rates are $13.50 per proof gallon of distilled spirits (i.e., the amount of liquid that contains one-half gallon of pure alcohol), $18 per 31-gallon barrel of beer, and from $1.07 per gallon of table wine to more than $3 per gallon for wines with higher alcohol content and for sparkling wines. In 1994 Federal Government revenues from alcohol taxation were $7.8 billion. In addition, State and some local governments also tax alcoholic beverages at various rates and collected a total of $3.7 billion from excise taxes on alcoholic beverages, plus an additional $4.7 billion from general sales taxes on these purchases in 1994. On average, Americans annually spend $64 per capita on Federal, State, and local excise and general sales taxes on alcoholic beverages (Distilled Spirits Council of the United States 1995; U.S. Bureau of the Census 1995). This amount does not include State and local government revenues from license fees or the net profit of State store systems, although these items could be considered taxes as well.

**Figure 2:**
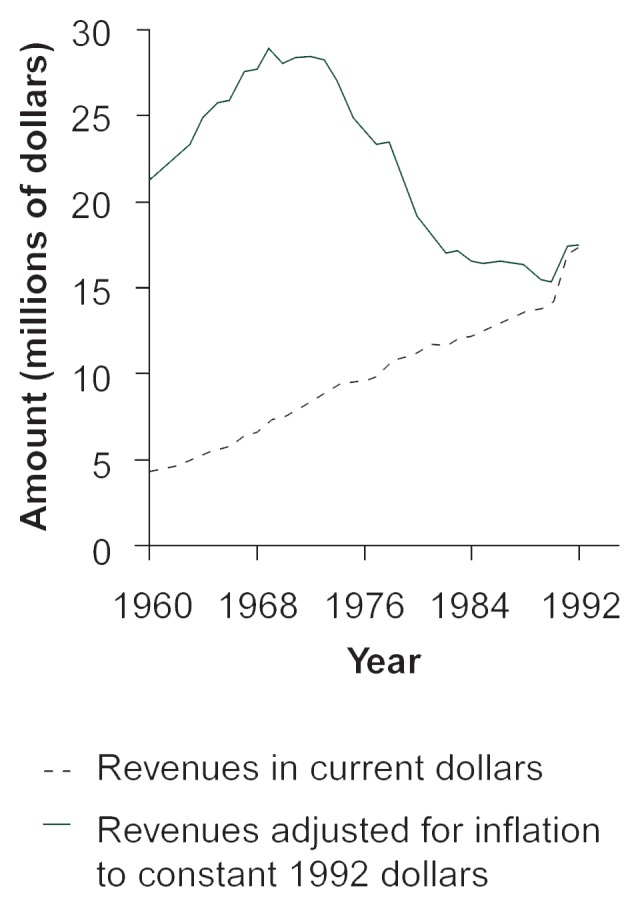
Total Federal, State, and local public revenues from excise taxes on alcoholic beverages, 1960–1992.

**Figure 3:**
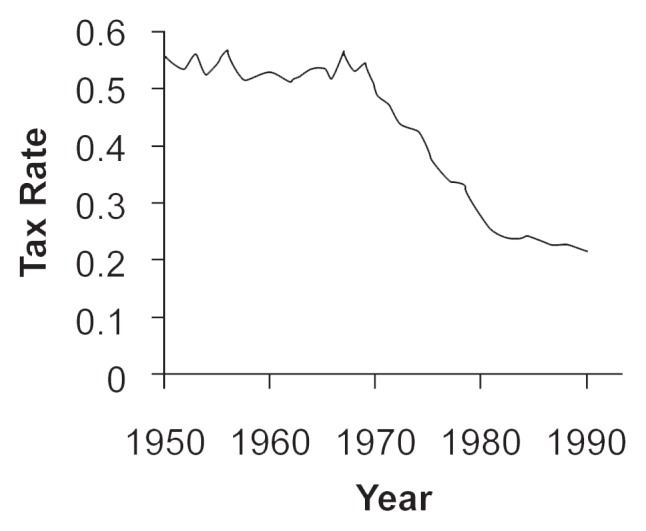
Average alcohol tax rate, 1950–1990.

Although the amount of actual revenues collected (in current dollars) has steadily increased from 1960 to 1992 (see figure 2), general price inflation makes comparisons of revenues measured in 1960 dollars and those measured in 1992 dollars misleading. When expressed in constant 1992 dollars, inflation-adjusted public revenues from alcoholic beverages increased somewhat from 1960 until the early 1970’s but fell steadily thereafter until 1990. Figure 3 shows the average alcohol tax rate over the same time period. In 1954 the average tax (including Federal, State, and local taxes) was more than 50 percent of the price of alcohol excluding the tax amount (i.e., the pretax price). (For example, if a consumer paid a total of $10 for a bottle of wine in 1954, the price might include $3.50 in excise taxes, which would compose about 54 percent of the wine’s pretax price of $6.50.) By the early 1980’s, the average tax had declined to less than 25 percent of the pretax price, because inflation eroded the real value of excise taxes given as a fixed amount. Thus, whether considering either inflation-adjusted revenues or average tax rates, the United States taxes alcoholic beverages less heavily now than it has for most of the postwar period. Although recent increases in Federal and many State excise tax rates are partly reversing this trend, some observers advocate indexing alcohol taxes to the inflation rate.

To put alcohol taxes in perspective, however, they currently are not a major source of revenue for either Federal or State Governments. For comparison, although States collected $3.7 billion in alcohol tax revenues in 1994, their revenues from tobacco taxes and State lotteries during the same year were $6.2 billion and $10 billion, respectively. At both the Federal and State levels, alcohol taxes account for less than 1 percent of total revenues collected (U.S. Bureau of the Census 1995).

Although alcohol tax hikes seem attractive in the current fiscal environment, they would not necessarily increase tax revenues. Whether total tax revenues would rise if tax rates were to increase would depend on the elasticity of demand. At one extreme, if alcohol demand does not respond at all to price (i.e., if it is perfectly inelastic), the quantity consumed would not change when prices rose; in this scenario, a 10-percent tax-rate increase also would increase revenues by 10 percent. In contrast, if alcohol demand responds at least somewhat to price, a 10-percent tax hike would increase revenues by somewhat less than 10 percent, because the quantity consumed would dwindle as the price increased to accommodate the additional tax. If alcohol demand is very responsive to price, the quantity consumed would shrink so much that revenues would actually fall after a tax-rate increase.

The best estimates to date imply that a 10-percent increase in the alcohol tax rate probably would increase the amount of tax revenues collected, although by less than 10 percent. A more precise estimation is difficult to calculate, however, because no consensus exists on exactly how responsive alcohol demand is to price, as noted previously. Many estimates of the price elasticity of demand for alcohol range from 0 to −1.0, indicating that alcohol demand is not highly responsive to price, although a handful of studies indicate the opposite (Leung and Phelps 1993). Nevertheless, based on the preponderance of evidence, a tax-rate increase seems unlikely to cause tax revenues to fall. In broad terms, this prediction is consistent with historical patterns: Inflation-adjusted tax revenues were high when the average tax rate was high and began to fall after the tax rate fell (see figures 2 and 3). Many other factors also influence alcohol consumption (and, consequently, tax revenues), however, including income growth, population growth, and changes in the population’s age composition (Nelson in press).

An interesting tension exists between the public health and revenue generation perspectives on alcohol taxation. Increasing alcohol tax rates would generate the greatest revenue if consumption is unresponsive to price—but if consumption is unresponsive to price, then alcohol taxation would not be an effective public health measure, because it would not reduce the quantity of alcohol consumed. Likewise, the more that alcohol tax hikes reduce consumption and alcohol-related problems, the less revenue they would generate. In practice, because alcohol demand appears to be price responsive to a certain degree, alcohol taxation retains some appeal as both a public health measure and a revenue generator. As Manning and colleagues (1995) note, however, their evidence that the very heaviest drinkers may be unresponsive to price suggests that higher taxes on alcohol could generate substantial revenues without as much of a negative effect on very heavy drinking as some members of the public health community would like.

## Alcohol Taxation From an Economic Efficiency Perspective

One of the primary policy concerns of economics is promoting efficient uses of the finite resources available to society. The economist’s view of efficiency is one in which society (i.e., the economy) produces the “right” mix of goods and services (i.e., outputs) with the “right” mix of capital, labor, and supplies (i.e., inputs) for the “right” group of consumers. Here, “right” means that it is not possible to reallocate the inputs, change the outputs, reassign the inputs so that there will be more output, or make some consumers happier without harming other consumers. Under certain conditions, a market-based economy can achieve this “efficient” mix of inputs and outputs. These conditions include the following:

- The markets for goods and services are perfectly competitive (i.e., a sufficient number of sellers offer goods and services so that no monopolies or imperfectly competitive markets exist).
- Consumers are well informed about the consequences of their activities.
- Consumers pay prices that fully reflect the costs of their actions to others.

The last condition—paying the full costs—is of concern when considering alcoholic beverages from an efficiency point of view. To understand how this condition affects efficiency, first consider another example: If a consumer buys an apple or a towel, he or she usually pays enough to divert all of the resources (i.e., capital, labor, and supplies) necessary to produce, distribute, and set that apple or towel apart from all other potential uses for the same resources and from other consumers. In other words, the consumer values the apple or towel by at least enough to bid the resources away from other activities (e.g., the production of cars) and from purchasers to whom the apple or towel has less value. In theory, competition among firms drives them to produce goods and services that command a market price sufficient to cover the costs of production and distribution and drives production cost to its lowest possible level using a combination of resources. Thus, the market process redirects resources from less valued to more valued activities.

Society maximizes the use of available resources only when the consumer pays for the full resource cost of the good or service and all its side effects (i.e., the full social cost). As a result, anything that drives a wedge between the full social cost of a good or service and the price consumers pay leads to an inefficient allocation of resources. For example, prices that are too low (e.g., because of explicit or implicit subsidies) or too high (e.g., because of monopoly or cartel pricing) both lead to inefficiency. If consumers pay less than the full cost of an activity, they divert resources from more valued uses to less valued ones, and if consumers pay more than the full cost, then either the amount consumed or the number of consumers could be increased while still covering the full costs.

In the case of alcohol, inefficiency may arise because the price paid by consumers is too low relative to the full social costs of drinking. Although the price paid for the beverage itself may reflect the costs of its production, distribution, and sale, the price may not be high enough to cover all of the costs (including the side effects, or “external costs”) involved in drinking. For instance, if a drinking driver has an accident that otherwise would not have occurred, then he or she pays for the beverage, plus any damage done to him- or herself and to property that is not covered by risk-adjusted insurance. The driver may not fully pay for the damage or inconvenience caused to others, however, such as the deaths and injuries of innocent bystanders. Thus, a problem drinker may pay only part of the full social costs incurred by his or her drinking. The price of alcohol is too low to reflect full social costs, because it fails to include the costs to others that result from a drinker’s alcohol consumption. As long as consumer demand for alcohol responds to price, a price that is too low will encourage increased consumption and associated problematic behavior and negative effects on others, thus creating a gap between the full social costs of drinking and the price paid by consumers.

### Use of Alcohol Taxes To Eliminate Inefficiency

Taxes can close this gap, because adding or increasing a tax on alcohol will raise its full purchase price. Furthermore, a higher price will motivate price-responsive consumers to reduce the amount they drink and, consequently, change their alcohol-related behavior. Theoretically, if the magnitudes of the adverse effects that drinkers have on others could be quantified, then analysts could determine a tax just large enough to offset the shortfall between private retail prices—which cover the costs of production, distribution, and sale of alcoholic beverages—and the full social costs of drinking.

Manning and colleagues (1989, 1991) estimated that the prevailing alcohol tax rates in the mid- to late 1980’s were about one-half of the amount necessary to make up the difference between private and full social costs of drinking; the chief contributor to the remaining gap was the loss of life by innocent bystanders in drinking-related accidents. The researchers’ calculation was incomplete, however, because it treated all drinking as causing uncompensated harm to others. A modified approach to determining an alcohol tax allows for the fact that not all drinking adversely affects others (Pogue and Sgontz 1989; Kenkel 1996). Even so, the revised tax still is higher than the current rates.

Ideally, society would tax (and hence, penalize) only problem drinking, because nonproblem drinking does not produce a gap between private and social costs. In practice, however, society taxes all alcohol consumption. Although an alcohol tax tends to bring the price paid by heavy or problem drinkers closer to the full social cost of drinking, a tax on nonproblem drinking leads to an efficiency loss, because it raises the price too high for nonproblem drinkers, thus decreasing their alcohol consumption. Adjusting for the differences between problem and nonproblem drinking results in a lower estimate of the “optimal” tax, but this estimate still is higher than the current tax rates for alcohol, especially for beer and wine (Blumberg 1992).

Such an adjustment relies heavily on knowing the price elasticities of alcohol consumption. The more responsive to price that nonproblem drinkers are, or the less responsive problem drinkers are, the lower the tax should be when considering the impact of taxing nonproblem drinking for costs it did not generate. If all else is equal, the less that nonproblem drinking changes when the price of alcohol increases, and the more that problem drinking is responsive to price, a tax will be an efficient way to address problem drinking.

### Policy Implications

Policy analysts considering economic efficiency select alcohol taxes that will reduce, but not eliminate, heavy or problem drinking. With such a tax in place, consumers willing to pay the full social cost still can choose to engage in heavy or problem drinking without imposing any net additional costs on the rest of society. In this scenario, the consumer decides what constitutes the “right” level of consumption according to personal preferences, because the benefits of drinking (as the consumer sees them) will just equal the costs of his or her actions to others.

Although this economic rationale results in an “efficient” pattern of alcohol consumption, it does not preclude harm to the drinker or to others. In contrast, public health advocates may want to continue to raise alcohol taxes as long as such tax increases reduce alcohol-related morbidity and mortality. As Cook and Moore (1993*b*) indicate, no upper limit may exist on the tax needed to improve public health. Even from the perspective of public health, however, very high taxes could be undesirable due to the potential health benefits of moderate drinking. To avoid discouraging moderate drinking, toleration of some problem drinking might be necessary as a trade-off. A key distinction between the economic efficiency and public health perspectives is that economic efficiency considers *all* the benefits of moderate drinking as seen by the consumer, not only the potential health benefits.

Alcohol taxation also has another efficiency aspect: Governments must raise revenues to cover expenses for defense, social programs, public works, and so forth, but how they raise that money can have serious efficiency consequences. In particular, whenever a government initiates or raises a tax, efficiency losses occur, because consumers must pay more than the cost of producing and distributing the taxed commodity. Depending on their degree of price responsiveness, some consumers will no longer buy the commodity, and many consumers will reduce their purchases as a result of the higher price. Such reductions lead to an efficiency loss.

Ideally, the government would place more taxes on goods or services with low price elasticities (Ramsey 1927), because the lower the price elasticity is, the less the reduction in consumption and the lower the efficiency losses. Thus, if the demand for alcoholic beverages were less price elastic than that for other goods and services, then alcohol would be a logical commodity to tax more heavily.

Sgontz (1993) concludes that the case for alcohol taxation is stronger when alcohol taxes are viewed as a substitute for taxes like the personal income tax. Personal income taxes generate economic efficiency losses, because they distort decisions between working versus enjoying leisure time; people may work less than they would if their labor earnings were not taxed.

## Alcohol Taxation From an Equity Perspective

Along with economic efficiency consequences, tax policies can have serious fairness (i.e., equity) ramifications to consider. One of the principles of equity is that people who are able to pay more should shoulder more of the costs of government and other services. Typically, this principle is manifested in the idea that the wealthier members of society should pay more than the poorer members, a concept referred to as “vertical equity.” In addition, if all else is equal, people in the same circumstances (i.e., in terms of wealth, opportunity, and so forth) should pay equal amounts, a guideline referred to as “horizontal equity.”

“Sin” taxes, such as excise taxes on cigarettes and alcohol, sometimes are criticized for falling more heavily on the poorer members of society. In the case of cigarettes, consumption diminishes (and, hence, paid taxes decrease) as income and education levels rise. In contrast, a number of studies seem to indicate that alcohol consumption (measured in drinks or volume) increases as income rises. The quantity consumed, however, does not increase as fast as income does. Thus, people with lower incomes still may pay more in alcohol excise taxes than vertical equity would dictate.

A sales tax on alcohol is somewhat different, because it is based on the amount spent on purchases rather than on the quantity purchased. Families in the highest one-fifth of the income distribution appear to spend a greater percentage of their income on alcoholic beverages than those in the lowest one-fifth, with intermediate values for the middle income groups (Sammartino 1990). Given that quantity consumed does not increase as rapidly as income, as previously noted, families may buy more expensive beverages as their income increases, not simply a greater quantity. If all income groups spent roughly the same proportion of their income on alcohol, then a sales tax on alcoholic beverages would be more fair than an excise tax that generated the same revenue, because richer families would pay nearly the same fraction of their income in alcohol taxes as would poorer families. If richer families spent more on alcohol than poorer families, however (as found by Sammartino 1990), a sales tax might even be progressive (i.e., the fraction of income paid in taxes increases with income), whereas the excise tax would tend to be regressive (i.e., the fraction of income paid in taxes decreases with income). Enthusiasm for the sales tax approach is dampened, however, by considering that a sales tax system at the retail level is much harder and more expensive to implement than an excise tax at the manufacturing level, especially if the tax rates vary across commodities.

An important note regarding equity is that a tax system may be inequitable for certain taxes but still relatively equitable overall. That is, the net effect of all taxes on all goods, services, and incomes may be relatively equitable, even though one component or another fails to meet the guidelines for either horizontal or vertical equity.

## Alcohol Taxation From an Employment Perspective

Another issue related to tax fairness concerns employment. Some people argue that alcohol tax increases will hurt workers whose livelihoods depend on the production and sales of alcoholic beverages. In 1994 the malt beverage, wines, and distilled spirits industries employed a total of approximately 56,000 workers (U.S. Bureau of the Census 1995), and liquor stores employed an additional 134,000. A tax increase that reduced alcohol consumption could be expected to reduce employment in these sectors. Typically, the affected workers would not come from the wealthier strata of society and would be in about the same circumstances as other people not hurt by the tax increase. Thus, a tax increase could violate the guidelines of both vertical and horizontal equity for workers in alcohol-related industries. An alcohol tax increase also could reduce employment to a small degree in other related sectors, such as eating and drinking establishments, which employ 6.6 million workers.

Overall, the level of employment in the United States is determined by macroeconomic conditions, however, not small adjustments in the tax rates on specific industries. When the national economy is not in a recession or depression, workers laid off or not hired by industries affected by an alcohol tax increase would find employment in other sectors of the economy. The distinction between job losses and worker displacement is crucial: A tax increase could cause a permanent job loss in the alcohol industry, but labor economics research suggests that the displaced worker almost certainly would find employment elsewhere eventually. Nevertheless, worker displacement remains costly during the spell of unemployment, as well as in the long run, because displaced workers appear to earn less on their new jobs. Using data from the Michigan Panel Study of Income Dynamics, Ruhm (1991) estimates that even 4 years after displacement, job losers earn 10 to 13 percent less than their nondisplaced counterparts. Jacobson and colleagues (1993) found larger earning losses for a group of experienced workers who separated from their firms in the early and mid-1980’s. Estimated earning losses also vary depending on the industry of employment, worker’s age, and local labor market conditions. For alcohol-related industries, the costs of worker displacement following an alcohol tax increase may be smaller or larger than costs estimated for groups of workers in other situations. Additional research focusing on alcohol-related industries could provide more reliable evidence on this point.

## Conclusion

Different perspectives on alcohol taxation lead to quite different conclusions about when an increase in an alcohol tax is either appropriate or effective. The substantial effects of problem drinking on others suggest that an alcohol tax could be used to reduce or eliminate what is, in effect, a subsidy to problem drinking. The presence of nonproblem drinking tempers this conclusion, however, and indicates the need to consider the price responsiveness of both problem and nonproblem drinking. For both economic efficiency and public health, a significant price response for problem drinking would allow shifts in alcohol taxation to be an effective public policy instrument. At the same time, a low price response for nonproblem drinking would enhance economic efficiency, because consumption by nonproblem drinkers would not decrease greatly after a tax increase and contribute to an efficiency loss. A low price response for all drinking would help satisfy the revenue generation objective, because the quantity of alcohol consumed would change little and would allow a tax increase to generate maximal revenues. Proposals to raise alcohol taxes also should consider the equity and employment effects that such an increase would have, however.

The current literature on the role of price and taxes in alcohol consumption and alcohol problems provides some of the information needed to inform choices about appropriate taxes, although estimates of alcohol price elasticity vary from one study to another. Further research is needed to examine the direct effects of alcohol taxes and prices across all patterns of alcohol consumption, especially problem drinking. In addition, quantifying the differences between the social and private costs of drinking requires more study.

## Footnotes

1 The term “alcohol abuse” in this article encompasses all levels of problem alcohol use and does not refer to a particular diagnostic system.

2 In this article, “problem drinker” and “problem drinking” refer to any type of alcohol use, even on a single occasion, that leads to adverse consequences in health, social, legal, or other life areas.

3 “Light,” “moderate,” and “heavy” drinking are not defined consistently in the alcoholism literature. In this article, the terms are used as they are given in the studies cited. Readers are encouraged to consult the original sources for precise definitions.

4 After adjusting for demographic and socioeconomic characteristics.

5 State-level aggregate data are often considered more reliable than microdata, because they contain objective measurements. The State-level traffic fatality rates examined in many studies, however, include fatalities not caused by drunk driving and exclude numerous cases of drunk driving that did not result in fatalities. Because microdata from surveys of individuals are not subject to the same types of measurement error problems as State-level aggregate data (Kenkel 1993), studying the price responsiveness of alcohol demand using this type of data can provide valuable information for policymakers. In addition, although self-reports of drunk driving are not perfect, they have been found to correlate well with more objective measures (Anda et al. 1987). The most prudent research strategy is to continue to analyze both aggregate data and microdata.
